# Rapid assessment of iron in blood plasma and serum by spectrophotometry with cloud-point extraction

**DOI:** 10.12688/f1000research.6716.1

**Published:** 2015-08-25

**Authors:** Tatyana Samarina, Mikhail Proskurnin

**Affiliations:** 1 Chemistry Department, Agilent Technologies Partner Laboratory - Analytical Centre, M.V. Lomonosov Moscow State University, Moscow, 119991, Russian Federation

**Keywords:** iron assessment, plasma, serum, cloud-point extraction, spectrophotometry

## Abstract

Rapid photometric assessment of iron in blood plasma and serum by a simple procedure after the extraction of iron(II) complex with 1-nitroso-2-naphthol in the micellar phase of a nonionic surfactant at the cloud point upon heating (pH range is 4.5–6.3) is proposed. The procedure trueness was verified using a standard reference protocol using bathophenanthroline. The advantages of the procedure are higher sensitivity than the reference protocol: the limit of detection is 0.03 μg/mL, the limit of quantitation is 0.1 μg/mL, the determination range is 0.1 – 2.8 μg/mL (RSD 0.02–0.10). Copper does not interfere with the iron assessment.

## Introduction

Iron level in blood plasma is affected by many physiological and pathological conditions ^1^. Plasma iron is determined in diagnosing hemochromatosis ^2, 3^, acute iron poisoning ^4^, active cirrhosis ^5^, or hepatitis ^6^, which lead to increased levels of transferrin, an iron(III)-binding glycoprotein that transports iron in the human body ^7^. Only 0.1% of the total iron is present in the blood plasma ^2^, thus its assessment should be rather sensitive, precise, and rapid.

Iron in plasma/serum is determined by spectrophotometry or atomic-absorption spectroscopy ^8, 9^ after the recovery of transferrin-bound iron(III) from acidic solutions using chelatants or detergents ^10^. Highly sensitive and specific though labour-extensive radioisotope ^11^ and immunological ^12^ assays for iron in blood plasma are seldom used due to the need for special equipment and expensive reagents. Spectrophotometric methods are most frequent in clinical practice and based on the formation of iron chelates with bathophenanthroline recommended as a reference method ^13– 15^ or its sulfonated analogue ^14, 16^, ferrozine ^17^, Ferene S ^18– 20^, or Chromazurol S ^21^. However, they are not always sensitive and (e.g. ferrozine) result in overestimation compared to bathophenanthroline ^22^.

We report rapid photometric determination of iron in blood plasma and serum by a simple procedure after the extraction of iron(II) complex with 1-nitroso-2-naphthol into the micellar phase of a nonionic surfactant at the cloud point upon heating.

## Methods

An Agilent Cary 60 spectrophotometer (USA; optical path length, 1 cm) and an inoLab pH Level 1 pH-meter (Germany) with a glass pH-selective electrode (precision ±5%) were used. Solutions were mixed with a Biosan MMS 3000 automixer with a micro-stirrer. Mass-spectrometry measurements were performed on a quadrupole Agilent 7500c ICP-MS (Germany) in a time-resolved analysis mode. The sample introduction system consisted of a robust Babbington nebulizer with a Scott spray chamber (Agilent Technologies) cooled by a Peltier element (2°C). The data were acquired and processed with ICP-MS ChemStation (version G1834B) software (Agilent Technologies).

A GSO 7765-2000 Russian certified reference sample of Fe(III) (1.00 mg/mL in 0.1 M HCl) was used for calibration. 1-nitroso-2-naphthol (Reakhim, Russia) purified as in 23, ascorbic acid (Fluka, China), neonol (AF-neonol 9–12, Elarum, Russia), sodium and ammonium acetates, HCl, trichloroacetic acid (all from KhimMed, Russia), bathophenanthroline (ReaKhim, Russia), and ethanol (Ferien, Russia) were used.

Buffer solutions (pH 4) were prepared by adding the necessary amount of a 1M sodium acetate solution to 0.1 M hydrochloric acid. Chemically pure chloroform (KomponentReaktive, Russia) pre-washed with water from hydrochloric acid was used as a micellar phase diluent.

Blood samples were provided by 2 healthy volunteers. All tests were made in 3 replicates. To obtain native serum, a sample was put in a clean glass test tube and left for 1 h at room temperature to form a clot. The clot was separated from the walls with a glass tip and the sample was centrifuged for 15 min at 1500 rpm. The resulting serum was transferred into a clean test tube. For the decomposition of the iron(III) complex with transferrin, 0.5 ml of serum/plasma in a glass test tube was mixed with 1 ml of 2M HCl, next, 1 ml of fresh 2.5% ascorbic acid solution was added. The sample was diluted to 5 ml and mixed thoroughly.

### Procedure with cloud-point extraction

A 1 ml portion of the test or a calibration solution is mixed with 1 ml of a 0.001M reagent solution in 5% neonol, 0.5 ml of 1M sodium acetate, and 8.5 ml of 5% neonol in a glass test tube. In the blank, 1 ml of distilled water was added instead of plasma/serum. Solutions were stirred in a boiling water bath for 15 min. Blood proteins denaturise and form a viscous white precipitate in the upper phase. Next, test tubes are cooled for 1 min in a cold-water stream, and the upper phase is removed by decanting. The lower, micellar, phase (0.6 mL) is diluted to 1.5 ml of chloroform and absorbance is measured at 715 nm against the blank.

### Reference procedure with bathophenanthroline

0.7 ml of the test sample was mixed with 0.1 ml of 1% ascorbic acid, 0.35 ml of 1M HCl, and after stirring, with 0.2 ml of 20% trichloroacetic acid and centrifuged at 1500 rpm. A 0.7-ml supernatant of the reaction mixture is transferred into a test tube, 0.6 ml of saturated ammonium acetate and 0.7 ml of a bathophenanthroline solution in ethanol are added. After 1 min, absorbance is measured at 536 nm against the blank.

## Results and discussion

The conditions for iron preconcentration with 1-nitroso-2-naphthol into a neonol micellar phase in the cloud point were selected as reported elsewhere ^24^. The iron recovery is 98 ± 2%. The optimum pH range is 4.5–6.3; the limit of detection is 0.03 µg/mL, the determination range is 0.1 – 2.8 µg/mL (RSD 0.02–0.10). The verification of the procedure using the reference protocol (bathophenanthroline) and an independent method (ICP-MS, isotope ^54^Fe) shows insignificantly different results ( Table 1).

**Table 1.** The results of extraction-photometric determination of iron(II) in biological fluids (concentration of 1-nitroso-2-naphthol, 1×10 ^-4^ M, pH 4.8, *t* = 15 min, *l* = 1.0 cm, *n* = 3, *P* = 0.95).

Sample	Blood plasma (µg/mL)	RSD	Blood serum (µg/mL)	RSD
**CPE procedure**	1.71 ± 0.24	0.06	1.32 ± 0.17	0.05
**Reference method**	1.74 ± 0.16 ^a^	0.05 ^a^	1.34 ± 0.29 ^b^	0.09 ^b^

^a^ ICP-MS

^b^ Reference protocol with bathophenanthroline

The own colour of the reagent does not affect the blank. It is noteworthy that copper(II), existing in significant quantities in plasma and serum ^25^, does not interfere with the determination as the absorbance maximum of copper complex with nitroso-naphthols lies at 430–490 nm. This avoids using toxic and corrosive thioglycolic acid as a masking reagent. In addition, sample procedure provides the denaturisation of proteins and their removal at the stage of phase separation. Finally, the advantage of the proposed procedure over the bathophenanthroline protocol is much higher sensitivity: while the reference protocol assumes the determination at the boundary of the spectrophotometer working range, the results for our procedure correspond to its middle. Moreover, the separation does not exceed 15 min, which is promising for the development of rapid assessment protocols. It is also noteworthy that the extraction occurs under rather soft conditions, and the pH interval of complex formation in the nonionic surfactant is rather wide ( *ca.* 1 pH both in acidic and alkaline ranges).

Dataset 1. Raw dataset for Samarina *et al.*, 2015 'Rapid assessment of iron in blood plasma and serum by spectrophotometry with cloud-point extraction' http://dx.doi.org/10.5256/f1000research.6716.d100757
Blood samples were provided by 2 healthy volunteers. All tests were made in 3 replicates. Click here for additional data file. Copyright: © 2015 Samarina T and Proskurnin M 2015 Data associated with the article are available under the terms of the Creative Commons Zero "No rights reserved" data waiver (CC0 1.0 Public domain dedication).

## Data availability

The data referenced by this article are under copyright with the following copyright statement: Copyright: © 2015 Samarina T and Proskurnin M

Data associated with the article are available under the terms of the Creative Commons Zero "No rights reserved" data waiver (CC0 1.0 Public domain dedication). 

*F1000Research*: Dataset 1. Raw dataset for Samarina *et al.*, 2015 'Rapid assessment of iron in blood plasma and serum by spectrophotometry with cloud-point extraction', 10.5256/f1000research.6716.d100757 ^26^
