# Supplementary material for: Rapid assessment of iron in blood plasma and serum by spectrophotometry with cloud-point extraction
Source: F1000Res. 2015 Aug 25;4:623. [Version 1] doi: 10.12688/f1000research.6716.1 (PMC4863669; doi:10.12688/f1000research.6716.1)
Supplement: Peer review history

# Peer review: Referee response for version 1

**DOI:** 10.5256/f1000research.7213.r13727

**Published:** 2016-05-09

**Authors:** Massoud Kaykhaii

**Affiliations:** 1 Department of Chemistry, Faculty of Sciences, University of Sistan and Baluchestan, Zahedan, Iran

This paper describes a method for the determination of iron in blood serum and plasma by a simple photometric procedure based on the cloud point extraction of a complex formed between ferrous ions and 1-nitroso-2-naphthol. Since spectrophotometric instrumentations own merits of simplicity, cheapness, portability and so on, this makes the paper interesting.

The authors claim that this is a “rapid” assessment of iron; therefore, it is better that the total analysis time be specified. Also it’s better to have the complex formation reaction between Iron (II) ions and the ligand. Moreover, I prefer to see a table in which a comparison between the proposed method with other spectrophotometry methods for the determination of Fe(II) in blood samples is included.

I have read this submission. I believe that I have an appropriate level of expertise to confirm that it is of an acceptable scientific standard.

# Peer review: Referee response for version 1

**DOI:** 10.5256/f1000research.7213.r12443

**Published:** 2016-02-22

**Authors:** Jean-Paul Canselier

**Affiliations:** 1 Laboratory of Chemical Engineering, INPT/ENSIACET, University of Toulouse, Toulouse, France

The authors should better specify the nature of the surfactant 'Neonol AF 9-12', that is a polyethoxylated nonylphenol with ca. 12 ethylene oxide units, and the conditions of the cloud point extraction. In fact, the surfactant cloud point is rather high (ca. 86°C?) and cooling down must be very sudden (quenching). Also, be sure that the conditions for iron preconcentration into the Neonol micellar phase are reported in ref.24.

In addition, the paper deals with complexation with 1-nitroso-2 naphtol whereas ref.24 describes complexation with 1-nitroso-2 naphtol-3,6-disulfonic acid.

The past tense ('was, were' instead of' is, are') should be used in the § 'Procedure with cloud point extraction' and 'Reference procedure with bathophenanthroline'.

I have read this submission. I believe that I have an appropriate level of expertise to confirm that it is of an acceptable scientific standard.
